# A Swarm-Based Approach to Generate Challenging Mazes

**DOI:** 10.3390/e20100762

**Published:** 2018-10-05

**Authors:** Joanna Kwiecień

**Affiliations:** Faculty of Electrical Engineering, Automatics, Computer Science and Biomedical Engineering, AGH University of Science and Technology, Al. Mickiewicza 30, 30-059 Kraków, Poland; kwiecien@agh.edu.pl; Tel.: +48-12-617-4320

**Keywords:** challenging maze, swarm intelligence, individual movements, maze complexity

## Abstract

Swarm intelligence draws its inspiration from the collective behaviour of many individual agents interacting with both one another and their environment. This paper presents a possibility to apply a swarm-based algorithm, modelled after the behaviour of individuals operating within a group where individuals move around in the manner intended to avoid mutual collisions, to create the most challenging maze developed on a board with determined dimensions. When solving such a problem, two complexity measures are used. Firstly, the complexity of the path was assumed to be a quality criterion, depending on the number of bends and the length of the path between two set points that was subjected to maximisation. Secondly, we focus on the well-known concept of the maze complexity given as the total complexity of the path and all branches. Owing to the uniqueness of the problem, consisting in the maze modification, a methodology was developed to make it possible for the individuals belonging to their population to make various types of movements, e.g., approach the best individual, within the range of visibility, or relocate randomly. The test results presented here indicate a potential prospect of application of the swarm-based methods to generate more and more challenging two-dimensional mazes.

## 1. Introduction

Based on the observations of the collective behaviour of the swarms of insects or herds of vertebrates, learning from each other and co-operating jointly for a common objective, a new field of computational intelligence was developed: swarm intelligence. The solutions occurring in natural systems, including the mechanisms that coordinate movement and work of a community, are also applied in many real problems. Most often, it is used to resolve the issues relating to optimizations, finding best routes, assignments, developing work schedules, task arrangement or object grouping. We should mention that the mechanisms of reaction to the incoming information and stimuli are based on simple rules. However, complete natural systems seem to be reliable and fitted with the capability of adaptation to environmental changes. The rules governing a swarm or herd were developed with time by instincts, and some of the rules were improved, owing to the individual agent’s ability to learn. A coordination system of a group of individuals, being a decentralised system, composed of autonomous units, is responsible for the organisation of tasks required for resolving a specific problem. Such a coordinated behaviour leads to the occurrence of dynamic material or social patterns at the group level, based on simple activities, interactions or feedbacks operating among particular individuals [1,2].

The natural adaptation of species and animals to the environment, and their ability to perform complex tasks have become the basis of various optimization algorithms. Well-known examples of such methods include ant algorithms [3,4,5] inspired by the behaviour of ants, particle swarm optimization algorithm [6] based on the social behaviour of bird flocking or fish schooling, and bee algorithms [7] imitating the foraging behaviour of honey bees in a swarm. Moreover, some algorithms in this category are the firefly algorithm [8,9], cuckoo search [9], cockroach swarm algorithm [10], krill herd algorithm [11,12], dolphin swarm algorithm [13], dolphin pod optimization [14], biogeography-based optimization [15], and many other methods.

It is of value to be aware that maze problems usually represented as grid-like two-dimensional areas lay the foundations for more practical problems, especially for network routing agents and autonomous walking robots, exploration or repair robots deployed in dangerous situations [16]. Note that the observation of spatial navigation capability and disorder in various mazes can provide a basis for better monitor the vehicles rout using the latest internet technologies. Recently, mazes were used to study artificial intelligence of robots by testing their capability to traverse unknown mazes [17]. A number of papers discussed various maze exploration algorithms. Present-day research projects concern the systems of autonomous robots, developed to be used in various situations [18]. Moreover, mazes, a good research paradigm for many navigation-based problems, have been used in the learning classifier system [19,20].

It should be mentioned that one of the best known approaches to maze generation is to apply graph theory. Each unit square constitutes a vertex and each path between two adjacent squares represents an edge. Consequently, edges exist only between the adjacent vertices. We should notice that we can pass to the adjacent vertex if there is an edge in between. Kruskal’s algorithm developed in [21] is frequently used for generating mazes. Another algorithm often quoted in the literature is Prim’s algorithm [22]. Moreover, Depth-First Search (DFS) is one of maze-generating algorithms: it is possible to build a maze upon assumption of a search space as a two-dimensional grid, with squares as vertices and passages to the adjacent squares as edges, as well as a random selection of the adjacent square to be visited. For a comprehensive description on DFS, see [23]. Another interesting method, which generates mazes, is an algorithm to construct vortex mazes with aesthetic effects [24].

Unlike swarm intelligence algorithms used to finding the shortest and most efficient route between two points, still little is known about these algorithms used to generate mazes. For example, an application of the basis of the cuckoo search algorithm to manage board games like mazes in terms of playability is presented in [25]. In turn, in [26], to compose of paths in the maze and to improve the efficiency of board games creation, two algorithms based on colony of ants and bees are used. In [27] an extensive survey on various bio-algorithms used to game creation and online management has been given. However, all these works are not intended to be used in creating the challenging maze with maximising complexity.

The goal of this paper is to present a method of generating complex mazes, with the use of the existing swarm intelligence mechanisms. We do not try to provide a new insight into the interactions occurring in the swarm. The aim of the paper is to propose a useful tool that deals with creating rectangular or square mazes, by using easily well-known mechanisms existing in swarms. The steps that lead to derivation of difficult mazes are outlined. It should be emphasized that by generating difficult and complex mazes, we are able to test robots in various maze configurations that use limited local knowledge, e.g., that obtained from sensors in real time, to plan the travel path.

Note that an open question is how to determine the appropriateness of parameters and the nature of movements individuals. With these comments in mind, the key objectives of the analysis are:

- to decide how different movements should be involved in the maze generation process to ensure the best chance of success for the algorithm,
- to avoid possible conflicts of movements that may arise,
- to assess parameters’ influence on the process of generating a maze.

Finally, our contribution addresses questions of how to implement a swarm-based approach with simultaneously maximising the complexity of regular mazes (in particular square mazes, with each wall as a straight line and an angle of 90 degrees between every two adjacent walls). The rest of the paper is organized as follows: Section 2 provides a short description of the swarm intelligence algorithms and a brief overview of their selected applications. In order to cope with their application to generate challenging mazes, we give more insight into considered problem in Section 3. Therefore, we present how to adapt the swarm intelligence algorithm to solve our problem, concerning the individual which represents a solution, movement performance and proper definition of typical parameters. Describing frameworks for swarm mechanisms applicability will provide a basis for seeking the best tool to solve such problem. In Section 4, the performance of the considered approach is tested and results of conducted experiments are presented. Finally, Section 5 contains concluding remarks.

## 2. Swarm Intelligence Algorithms and Their Applications

### 2.1. Swarm Intelligence Mechanisms

The swarm intelligence systems are based on a population of simple agents, locally interacting with both one other and their environment. Global behaviour and local interactions between the individuals become the basis of a number of calculation models used successfully in many fields. The main objective of swarm intelligence is to aggregate of individually behaviour and interacts with the adjacent agents and the environment in order to develop collective behaviour that can be useful for collective problem solving. The conflicts between the prey and the predator, contributing to the occurrence of the most important adaptations, and the individual’s ability to relocate itself actively allow us to recognise specifying animal behaviour. Similar navigation methods that are employed by animals when relocating in space, for example by bees using polarised light to determine the flight direction between the beehive and the areas abundant in nectar, or route marking with pheromones, as it is done by salamanders or ants, contribute to our better and better understanding of animal communities. Many bird species and some mammals live in small flocks, while such social insects as ants, bees, or termites create large communities. Such common life, within particular social structures, allow them to benefit in many ways, for example when hunting, searching for food, storing resources, defending against intruders, observing the surrounding areas, or upbringing the young. The young imitate the older individuals in the group or flock to acquire survival skills. When analysing the animal world, one can observe diverse sensibility of animal organs [1,2].

A number of algorithms was developed to use the analogies with natural systems in which the basic coordinating mechanisms include self-organisation, i.e., a set of dynamic mechanisms of the creation of behavioural patterns of a flock of individuals, with the use of various lower-level interactions, or stigmergy consisting in direct coordination of individual behaviour with the use of environmental modifications. Living organisms apply strategies to build social structures became the foundations of many algorithms as mentioned in the previous section. A comprehensive review of swarm intelligence methods can be found in [9,28].

Swarm algorithms involve the procedures concerning the individuals’ movement within the group. For example, in the firefly algorithm, the firefly movement is determined by several factors such as its current location, attractiveness and randomness. Each firefly moves towards a brighter partner [9]. In turn, in the cockroach swarm optimization (CSO) algorithm, the procedure consists in the individuals’ movement with the swarm towards that one that enjoys the best target function value, relating to visibility. If a given individual cannot see a better one, it moves towards the swarm leader. In that way, the locally best individuals create small swarms and follow the best one in the group. The individuals also perform random movements, reflecting the dispersion situation. That procedure allows them to avoid being stuck in a local optimum. Exact information regarding that algorithm can be found in [28] (pp. 232–233) and [29,30].

### 2.2. Selected Applications of Swarm Algorithms

The algorithms that are based on swarm behaviour are treated as promising tools for many applications, owing to their simple implementation, short computational time, and effective mechanisms that prevent the agents being stuck in their local optimum. The multiple applications of swarm intelligence also include optimization problem solving, finding the shortest route, assignments and scheduling, or object grouping. Owing to high flexibility in the adjustment to changing dimensions of solution spaces, swarm algorithms were used for solving the travelling salesman problem [29,31]. The paper [32] presents an automated guided vehicle (AGV) control system, using the Wireless Sensor Network technology so that the vehicles can operate as mobile robots, using the swarm intelligence to coordinate vehicle operation. The algorithms based on swarm intelligence are also applied in solving the Quadratic Assignment Problem (QAP), modelling a number of such issues as the travelling salesman problem, generalised problem of graph subdivision, or the problem of finding a maximum clique [33]. Certain studies also concerned the application of swarm algorithms to tune PID controllers [34,35,36,37]. Many researchers dealt with swarm-based approaches to train classifiers. In [38], particle swarm optimization and artificial bee colony were presented to train feed-forward neural network for pathological brain detection. Moreover, Wang et al. [39] employed real-coded biogeography-based optimization to find the optimal weights and biases of feedforward neural network optimization. In turn, in [40], a classification system for Alzheimer’s disease, combining wavelet entropy and multilayer perceptron optimized by biogeography-based approach was developed.

Some mechanisms that constitute the foundation of social structures can be used to control a colony of robots. Swarm Robotics (SR) is one of the most promising technologies intended to develop reliable, scalable, and flexible systems. Swarm intelligence mechanisms have been applied in many various tasks performed by a group of autonomous robots that are capable of moving and interacting with their environment, without a centralised control system [41]. For example, the situation that occurs in bird flocks or schools of fish, where individuals move in the direction of a known target location, avoiding collisions, is reflected in designing robot group relocation towards the selected target. “Foraging” or “area plundering” techniques are often applied in swarm robotic systems. Robots have to collect the objects acquired from their environment and bring them back to their nest. “Foraging” can reflect demining and search and rescue operations. The technique can be applied for collective land exploration, cargo transportation, or decision making, as well as for the purpose of testing the influence of disturbances on a group of robots [42].

In [43], swarm intelligence has been proposed for the management of autonomous and independent unmanned aerial vehicles (drones). The challenges faced by fleet of drones during all operations ranging from flight control to cybersecurity are investigated. It should be noted that drones can learn on the basis of the situation in which they find themselves. Besides, swarm intelligence mechanisms are being applied in Internet of Things applications, modern data networking, the efficient management of large-scale ad-hoc networks (wireless sensor networks) and so on [44,45]. In [46], comprehensive reviews for swarm intelligence based routing protocols for Wireless Sensor Networks were provided.

In summary, swarm-based algorithms are very useful in a diverse range of optimization tasks in real applications. A broad overview of various applications is presented in [47]. It should be mentioned that many issues exist concerning these approaches [48]. One of the challenges is the appropriate design and implementation of their all procedures. Note that the methods should assure a good balance between exploration and exploitation. The characteristic parameters of solutions, the settings of control parameters, and the type of movement mechanisms affect the quality of the algorithm. Recently, we have seen the progress towards the performance of swarm algorithms in the maze problems. As mentioned in the previous section, some experiments were done in the domain of board games. However, there is still a gap between these algorithms and creating the challenging maze with higher complexity.

## 3. Swarm Movements to Generate Challenging Mazes

Swarm algorithms perform well in a number of optimization problems, although they usually require the adaptation of certain mechanisms, especially those relating to the uniqueness of the movement being performed. Our approach is based on the cockroach swarm optimization algorithm [28] (pp. 232–233). The rationale of adopting this algorithm lies on simplicity of its concept and its potential applicability in some optimization problems. Generally, it is initiated with a set of the random solutions. In our case, each maze is represented as a “cockroach”. With every iteration, the search individual update its solution based on either the best solution obtained (within its visual scope or the best solution obtained so far) or a random movement. For example, in [29] we offer approaches to deliver ways of movements for the adaptation of the cockroach swarm optimization algorithm to the traveling salesman problem including crossover operators known from genetic algorithms and a 2-opt move. In turn, in [30] a procedure of individuals’ relocation had to be also developed to resolve the time-expanded graph in journey planning. As was mentioned in previous section, the observation of spatial navigation capability in various mazes can provide a basis for better monitoring the vehicles or robots rout. Therefore, we suggest a scenario in which the CSO approach manages conscious changes of mazes. At the same time there is the crucial aspect: how its mechanisms can enhance maze complexity and modify the solution. Note that the CSO used for our problem should be applied with more maze-specific modifications. Here, the way in which individuals move in generating a challenging maze is described in Section 3.2.

### 3.1. Problem Statement

Let our objective be to generate a challenging maze of *n*-by-*m* cells, in which there is a path from the entry point to the exit point lying at the other end of the maze. First, we define the top left corner as the entry point and the bottom right corner as the exit from the maze. We assume that our maze is a typical model, i.e., there is only one solution to reach the goal location. With the defined number of rows and columns, we select such a cell wall arrangement that will allow us to obtain the most challenging maze possible. The difficulties of moving within the maze involve dead ends and winding paths. All the corridors between the partitions are cell-wide. The maze does not contain any cycles, which means that one can arrive only once at one forking-path point, since multiple passing through the same corridor is not admitted. Note that the corridor is defined as a path between two cells, each of which is either the beginning, the end, a fork, or a dead end in the maze. Therefore, we can solve the path finding problem for example by Dijkstra’s algorithm [49].

**Figure 1:**
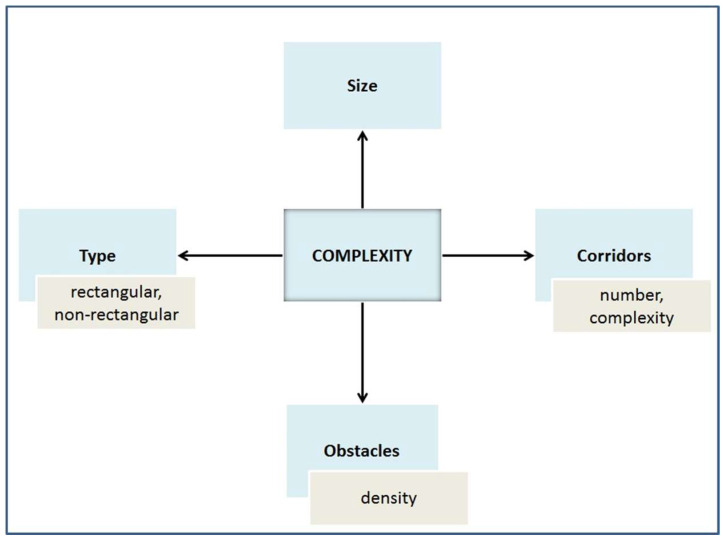
Factors effecting the measure of the complexity of mazes.

Now, we talk about the complexity of mazes. Note that the complexity measure of a maze M may be different and the measurement of complexity in mazes is a difficult problem [50,51]. Hence, it is of value to be aware of factors affecting the complexity of the maze, as illustrated in Figure 1.

The complexity of the maze depends on the number of cells that comprise the maze (related to the length of the path between the start and end points), the density of the obstacles, the number of corridors, and their complexity.

It is well note that the measure of the complexity of a corridor depends on existing turns. When a corridor alters quickly its direction, the measure of its complexity will be the higher. Therefore, complexity depends to a significant degree on topological and geometric properties of a given maze such as the length of the solution path, twistiness, and the number and length of the branches. In [20], new metrics for classifying the complexity of mazes based on agent-independent and agent-dependent characteristics were introduced. As we can see, there are different ways in which the measure of the complexity can be calculated. A more in-depth discussion on conducting a complexity analysis can be found in [50,51].

Now we turn to the complexity of regular mazes used in our approach. Let *h* be a corridor with two endpoints and *T* be a solution of a maze *M* as the path of an individual moving between the entry and exit points. If the direction differs from the current direction by 90° (either to the left or to the right), then we say that a corner point belonging to the corridor *h* exists.

As mentioned above, depending on factors affecting the complexity of the maze, different maze complexity metrics can be depicted. Therefore, we consider two different variants for complexity measures.

#### 3.1.1. Criterion “the Complexity of the Path”

The first one, named by us as “the complexity of the path”, involves assumption that the measure of the complexity of mazes will depend on the complexity of the path (*T*). Suppose *D*(*T*) is a the total length of the path and *no_cp* is the number of its direction changes. Therefore, the maze complexity *γ*(*M*) of *M* is given as:

(1) γ(M)=D(T)·no_cp

#### 3.1.2. Criterion “the Complexity of the Path and Branches”

As is known, maze complexity increases as the complexity of *h* increases. On the other hand, if the measure of the complexity of mazes depends on the total length of the path and the number of its direction changes, the solution to that objective function could be envisioned. In order to avoid this possible issue, we also consider the second metric given in the literature (named by us as “the complexity of the path and branches). We focus on related work in complexity [51]. Therefore, we assume that the main determinants of the maze complexity are: total length of the path between the entry and exit points, the alternative (dead end) path lengths and turns.

Formally, we have a set of cells belonging to *h* (called as *W_h_*). We consider the complexity *γ*(*h*) of *h* from two endpoints *k* and *l* by [51]:

(2) $$\gamma \left(h\right)=D\left(h\right)\sum_{i=1}^{k}\frac{\theta \left(w_{i}\right)}{d\left(w_{i}\right)\cdot \pi }$$

where:

- *D*(*h*)—the length of *h*,
- *d*(*w_i_*)—the length of *h* between *w_i_*_−1_ and *w*_i_ belonging to *W_h_*,
- *θ*(*w_i_*)—the absolute value of the difference between the current and previous direction (in the radian measures).

We assume that if each new branch changes its direction from the path or previous branch (in relation to the path or the previous branch from which it leaves), *θ*(*w_i_*) will be equal to *π*/2.

Taking into account the set of branches *B* = {*B*_1_, …, *B*_n_} and the set of all hallways in some branch *H* = {*h*_1_, …, *h_n_*}, the complexity *γ*(*M*) of the maze *M* can be defined as follows [51]:

(3) $$\gamma \left(M\right)=log\left[\gamma \left(T\right)+\sum_{i=1}^{n}\gamma \left(B_{i}\right)\right]$$

where the complexity *γ*(*B_i_*) of some branch *B_i_* computed as a sum of its all hallways complexities.

### 3.2. Proposed Methodology to Generate a Challenging Maze

As we know, the first stage of each swarm intelligence algorithm is to generate the initial population of solutions. This process is randomly carried out by the application of the depth-first search (DFS) algorithm [23]. At the beginning, each cell has four walls, and the algorithm starts randomly. Then, all neighboring cells of a given cell are tested, followed by the return to the cell from which the current cell was visited. A random unvisited neighboring cell is selected and the wall between these two cells is removed. Such a new cell is denoted as visited. It is worth pointing out that all transitions between cells are generated in the way to avoid cycle generation (for example by building new walls).

We assume that the solution quality is determined by maze’s complexity. Therefore, upon generation of initial solutions, their quality is estimated. The purpose of the subsequent steps is to improve solutions and find better maze with higher complexity.

In the swarm movement procedure (known as chase-swarming procedure in the cockroach swarm optimization algorithm), movement was determined by partial replacement of the path constituting the solution of the given maze. The path was recorded in the form of a vector containing the numbers of the related vertices. During the swarm movement procedure, first, the common points of both vectors are determined. In the vector corresponding to a weaker individual, there occurs a replacement of elements for those originating from the vectors corresponding to a stronger individual, between two randomly selected common points. Once a maze is remodelled, the corridors relating to the specific path element will also be supplemented. Once those operations have been completed, it is necessary to check whether some corridor elements are not cut off (they should be rather connected to the closest corridor) or some clusters are not filled with walls or empty spaces (corridors are created randomly). As a result of such replacement of some of the path and corridors, one can be faced with the situation where there is more than one solution. If that is the case, all the corridors creating a solution will be broken (except for the defined path). Such a mechanism ensures maze modification, without creating cycles or inconsistent components. What is essential in this procedure is a proper interpretation and determination of the visibility parameter. We assume that the visibility parameter (*visual*) denotes the minimum number of common cells (in the vectors that are solutions of the two mazes) that two individuals should have in order to be visible to each other. Note that too high value of that parameter will cause that the individuals will not group in small swarms, and certain potential solutions will be omitted.

Similar mechanisms are used in the individual random dispersion procedure. For each of the individuals, a new individual is created randomly (let’s call it a temporary individual). The individuals will move in the direction of their temporary individuals, regardless of the fact whether those reflect harder or easier mazes. Since the dispersion procedure implements a considerable changeability of mazes, currently the best solution (of all the previous iterations) should be remembered.

The searching procedure of the best maze ceases if the stopping criterion is met, e.g., the maximum number of iterations or number of unimproved iterations. The foregoing operations of the presented approach are outlined as Algorithm 1:

**Algorithm 1****initialize:**  maze’s parameters: *n*, *m*  parameters: population size (*k*), visual scope (*visual*), maximum number of iterations (*MaxIt*)  randomly population of *k* individuals (each individual is one generated maze)**for**
*i* = 1 to *k*
**do**  evaluate the quality of its solution (the complexity of the maze)**end for**find the best complicated maze (*P_g_*) in initial population**while** iteration < *MaxIt*
**do** **for each** individual *i*
**do**             // start procedure: *swarm movement*  **for each** individual *j*
**do**    **if** the complexity of individual *j* is higher than the complexity of individual *i*, within its    *visual*
**then** move *i* towards *j*; **end if**    **if** the complexity of the *i*th maze is a local optimum (within its *visual*) **then** move *i* towards *P_g_*;    **end if**  **end for** **end for** **if** the new solution is better than *P_g_*
**then** update *P_g_*; **end if** **for**
*i* = 1 to *k*
**do**           // start procedure: *random dispersion*  move the *i*th individual towards its temporary solution **end for** **if** the new solution is better than *P_g_*
**then** update *P_g_*; **end if****end while**return the most complicated maze with the highest measure of complexity

In order to understand the architecture of our approach, it is necessary to present the individual movement. Therefore, this step of implementing the individual movement (the individual moves in the direction of its temporary solution or tends toward another individual) can be stated as follows:

***STEP: the individual movement*****if** the number of common points in the path is greater than *visual*
**then**
randomly select two common points;change the elements of the path vector of the *i*th individual into elements of the path vector of the *j*th individual between common points;move (from *j*th to *i*th individual) the corridors reaching the replaced segment of the path
**if** there are cut-off corridors (not connected with the start or end points) **then** add the cut-off corridor to the nearest corridor;**end if****if** there is a solution different from the set path vector **then** introduce changes to the maze structure by replacing a randomly selected element not belonging to the path vector with a value corresponding to the wall;**end if****if** four elements corresponding to the walls are concentrated in the maze **then** replace one randomly selected element in a cluster of these walls to the value corresponding to the corridors;**end if****end if**

## 4. Experiments and Results

Let us examine several results of the conducted experiments in order to test the effectiveness of the implemented algorithm. We assumed that all mazes had regular shapes. Although we carried out many experiments for different size of maze, we report our results for square mazes. Moreover, the maze has two free cells: start and target, and all the others are unknown. The algorithm was implemented in the Matlab programming language, using a Windows 8.1 operating system on an Intel® Core™ i3-4000M 2.40 GHz processor equipped with 8 GB RAM. We explore two different variants of the complexity objective function.

### 4.1. Experiments for the Complexity of the Path

The first step in our research is the determination of the complexity of a maze in relation to the population size in the operation of the presented swarm-based approach. Therefore, the selected results will be discussed with a view to characterizing the information content resulting from the algorithm’s run. In what follows, we will restrict attention to the results obtained for the 25-by-25 maze. In all experiments, we assumed *visual* = 10 during all iterations. Moreover, the maximum number of iterations was 40. The population size was set to 5, 15 and 25 respectively. It is important to note that these experiments were done using other fixed parameters during all iterations. The best values of maze’s complexity obtained during selected runs, depending on the population size, are gathered in Table 1.

Table 1 is organized as follows: the first column contains selected run of our approach. The columns “Initial”, “Output”, and “Number” contain information on the value of the complexity of the best initial maze, the highest complexity of a maze obtained through each run of our approach, and the iteration number with the best solution, respectively. The last column “Improve” shows the improvement of the results obtained by mentioned method. It needs to be pointed out that such an improvement was calculated by dividing the difference between the results of the “Output” and “Initial” columns by the corresponding results of the “Initial” column, then multiplying by 100%.

**Figure 2:**
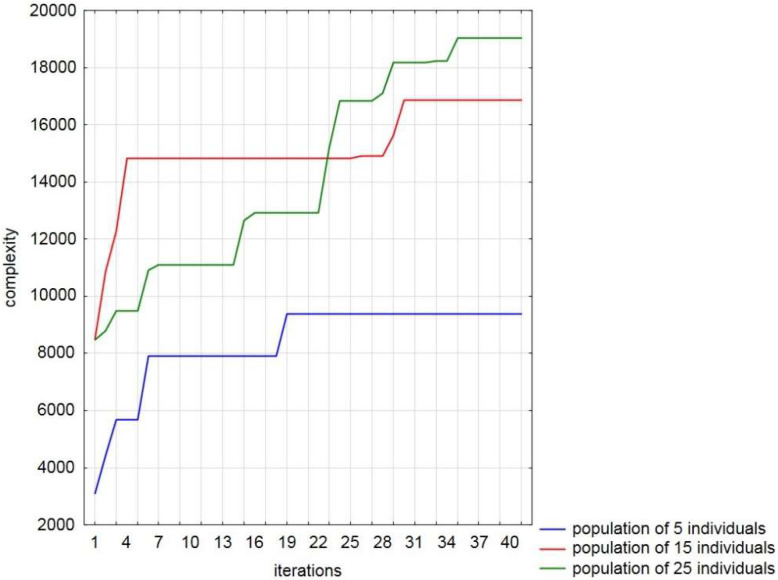
The best run of the presented method for generating mazes with the highest measure of complexity during 40 iterations, for various sizes of the population. The vertical axis is the value of complexity, and the horizontal axis is the number of iterations.

A selected run process of the implemented algorithm for the maze of 25-by-25 cells is illustrated in Figure 2. We have seen the progress towards higher complexity with our algorithm, relating to various sizes of the population. The results show that more individuals in the population improves its ability to explore the space of solutions and find mazes with higher complexity. Among the results obtained through 100 independent runs of our method, the complexity of the maze amounted to 19,039 was found for the population size equalling 25 solutions (see the first trial in Table 1). It needs to be pointed out that for this solution it yields an improvement about 124.6%. The obtained statistics over 100 runs of the algorithm are summarized in Table 2. It displays the best and worst solutions found by the implemented algorithm (columns “max” and “min”, respectively) for the complexity and improvement measures, the average solutions of 100 independent runs (column “average”) and the standard deviations of the found solutions (column “STD”). As one can see, in all cases we have the percentage relative improvement between the best value of the objective function value found by the algorithm and the best initial value.

As expected, the increasing population improved the chance of finding the most complicated solution over all runs for the test maze. Our tests showed differences of the results obtained between the population size equalling 25 individuals (the average improvement was 241.09%) and other sizes. Therefore, one of the findings of our preliminary study is that increasing the population size seems to improve the performance of the algorithm.

**Figure 3:**
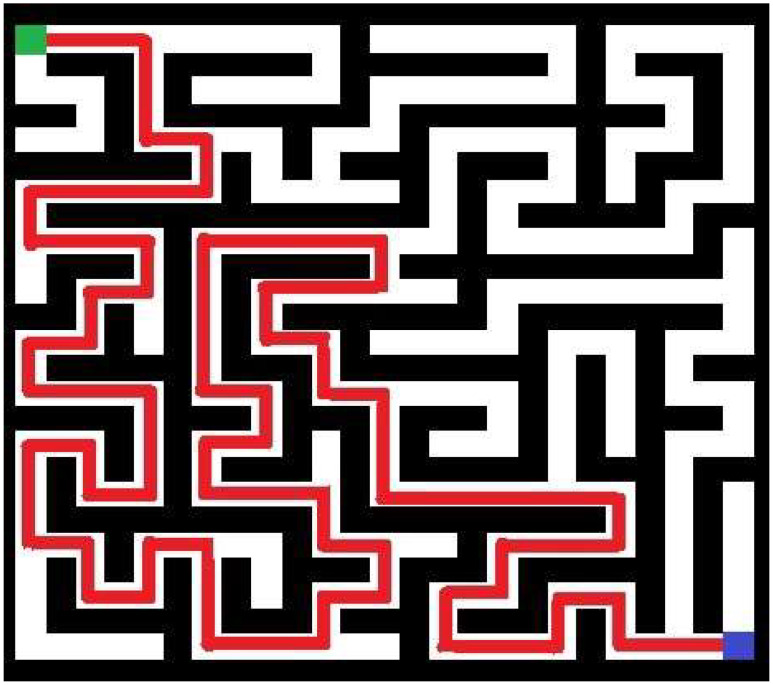
The maze in the initial population of 15 individuals with the highest measure of complexity (green represents the start point, blue denotes the exit).

**Figure 4:**
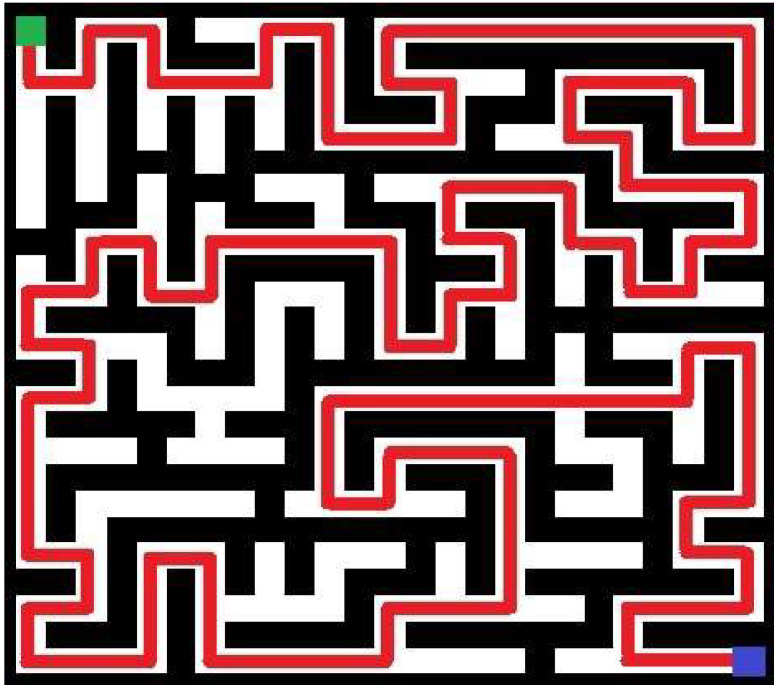
The maze with the highest measure of complexity after 40 iterations for a swarm of 15 individuals (green represents the start point, blue denotes the exit).

In turn, in Figure 3 above, the best individual in the initial population of an example run of the implemented approach is shown. In this particular trial (the fifth trial in Table 1), a swarm of 15 individuals was employed to generate the more complicated maze of 25-by-25 cells. The maze with the highest value of complexity obtained during 40 iterations in the same run is shown in Figure 4. Referring to Figure 3 and Figure 4, generated mazes have complexities of 8478 and 16,875, respectively (an improvement is equal 99.05%). Note that mazes are shown in terms of blocked (black) and accessible (white) cells. The path for these mazes is marked as red line. Green and blue squares denote the entry and exit points, respectively.

When analyzing the results obtained with the use of the algorithm, we should notice that the increase of the population size increases the number of function evaluations in each iteration. Therefore, Table 3 corresponds to a comparable number of function evaluations (1000 during the whole run of the algorithm).

**Figure 5:**
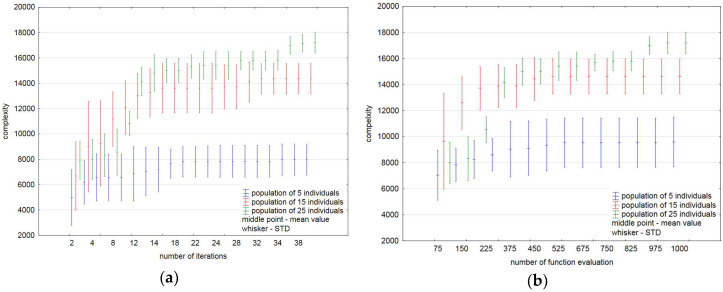
Mean and standard deviation values over 100 independent runs: (**a**) during 40 iterations; (**b**) with a fixed budget of function evaluations.

The statistics obtained over 100 independent runs for the test maze indicate that the variability of the results obtained is inevitable. Figure 5 shows the average (mean) and standard deviation (STD) values of complexity changing during 40 iterations (Figure 5a) and the number of function evaluations equalling 1000 (Figure 5b).

It is clearly visible that for a small population (five individuals in our case), the increase of the number of iterations and so the number of function evaluations leads to a slight improvement in the results obtained. One can see a very small improvement in the average value of complexity for the population size of 15 individuals.

### 4.2. Experiments for the Complexity of the Path and Branches

What if we take the second objective function (see Equation (3))? The motivation for using this function is that it is more complicated and the swarm algorithms can result in a lack of improvement. We still use the same way of movement as we used before. Note that the basic instance that we consider in this work is the maze size of 25-by-25 cells. Recall, the control parameters of our approach can influence the results obtained and the algorithm behavior. Therefore, experiments were conducted and repeated to determine how selected parameter values influenced the degree of complexity of the generated maze. Moreover, a result is kept of how the number of iterations it takes to build mazes with higher complexity on each run.

**Figure 6:**
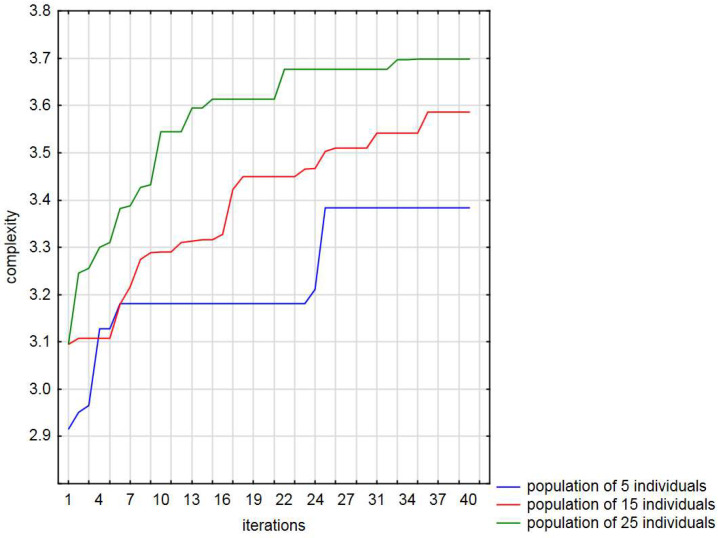
Comparison chart of the best run of the presented method for various sizes of the population during 40 iterations. The vertical axis is the value of complexity, and the horizontal axis is the number of iterations.

Figure 6 shows the results of the selected run process for the second objective function, for the population size ranging from five to 25 solutions. As can be seen, a fairly significant improvement of the optimization process occurs upon increase of the population size. The approach was able to improve the results in all of the trials. Firstly, each run of the presented algorithm was terminated after 40 iterations. The best result was obtained for the population equal to 25. Note that the best initial solution for said population size was the same as the best solution for the population equalling 15 individuals. Based on this optimization process, it is also clear that an increasing in the number of solutions made a considerable contribution to the challenging maze creation and yielded an increase in complexity. When analyzing the results obtained with the use of the algorithm (see Table 4 and Table 5), we can notice that the best improvement of complexity is equal to 19.48%.

**Figure 7:**
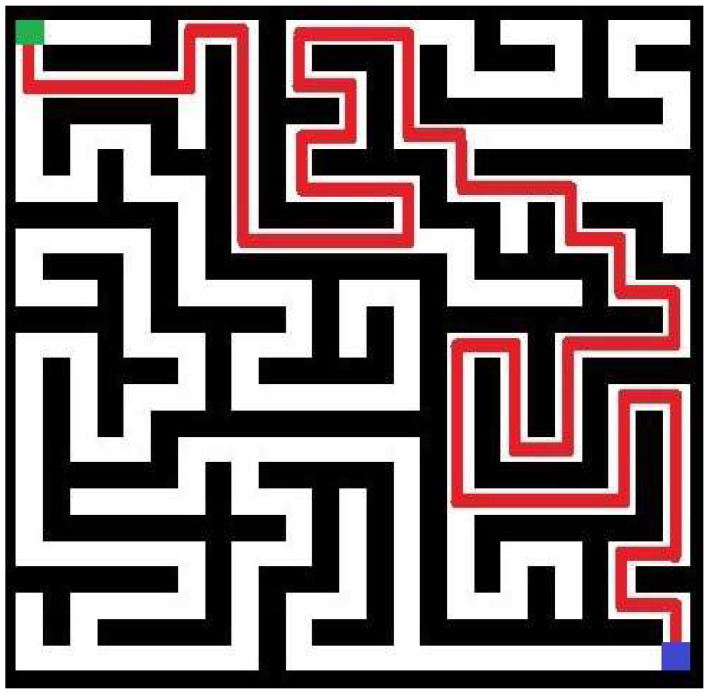
The maze in initial population with the highest measure of complexity (green represents the start point, blue denotes the exit).

**Figure 8:**
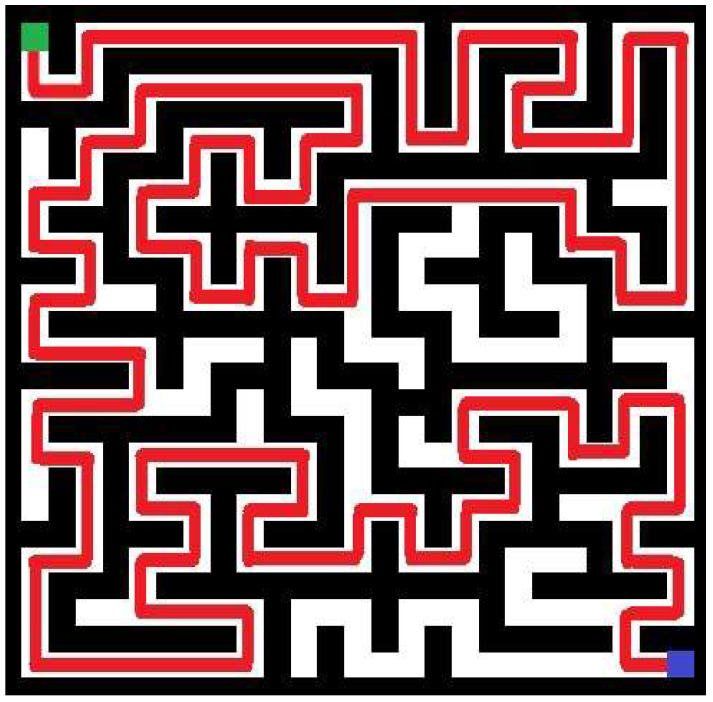
The best maze after 40 iterations (green represents the start point, blue denotes the exit).

It needs to be pointed out that this improvement of the maze complexity (calculated by dividing the difference between corresponding the best final and initial results by the best results of the initial solution, and multiplying by 100%) is on a logarithmic scale. Figure 7 shows the best maze in the initial population, while Figure 8 shows the best result of the implemented algorithm in this particular trial. These mazes have complexities of 3.0951 and 3.698, respectively.

**Figure 9:**
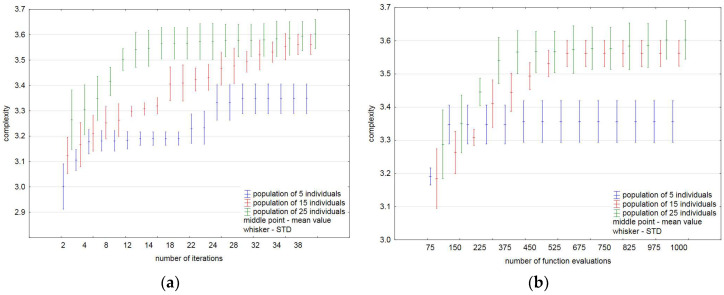
Mean and standard deviation values over 100 independent runs: (**a**) during 40 iterations; (**b**) with a fixed budget of function evaluations.

As shown in Table 4 and Table 5, the largest standard deviation over 100 independent runs is for the population of 5 individuals, after 40 iterations and the number of function evaluations equalling 1000. However, it should be noted that if the number of functions evaluations is taken into account, the results obtained for the population of 5 and 15 individuals are slightly better. Such a small improvement in relation to a fixed number of iterations (for all populations) comes from the fact that this algorithm shows the greatest convergence at its beginning. Figure 9a,b show the selected statistics of the results associated to 40 iterations and the number of function evaluations, respectively.

Above 40 iterations, this improvement occurred only a few times during all experiments. It should be mentioned that the best results obtained were worse in most runs.

### 4.3. Summary of Experiments

As mentioned in previous sections, our aim was to show how challenging mazes could be obtained with the cockroach-based approach. We conducted many experiments for two objective functions (the complexity of the path, and the complexity of the path and branches) and presented the results obtained over many runs of the implemented algorithm, including statistical analysis of the results. The optimization process was illustrated for a user-specified number of iterations or function evaluations. For these considered objective functions, we carried out preliminary research on the impact of population size on the obtained values of the complexity of mazes. The algorithm achieved the worse results with taking into account the complexity values for a population of 5 individuals for both objective functions. Clearly better results were obtained for the population size equalling 25.

It should be mentioned that the increase of the number of iterations and so the number of function evaluations provided a slight improvement in the results obtained. In summary, the total number of function evaluations can only be a preliminary indication to determine the appropriate number of iterations due to the population size. Therefore, we cannot assume that the same number of function evaluations in the case of different sizes of the population will allow us to achieve the same results.

## 5. Conclusions

In the paper, we have presented a swarm-based approach for constructing challenging mazes (2D) that are difficult to solve. We show how to obtain a difficult maze with selected dimensions and an increasing degree of complexity, by application of individual’s movements in the swarm. The algorithm could support maze design across a variety of sizes. Note, a bigger size of a maze increases the chance of its higher complexity. The challenged mazes could be created to mirror the obstacles devices face in Internet of Things environment. For example, such solution could be used to examine the efficiency of a sensor-tagged vehicle as it travels through mazes.

As shown in the paper, swarm algorithms can be used to modify mazes in order to increase their complexity level. However, it is necessary to apply a properly determined procedure of movement performance, so that the newly generated maze fulfils the assumed requirements (single solution and lack of wall and empty space concentrations). Based on the results, it is possible to draw the following conclusion: the lower the value of the population size parameter the worse results are obtained. The results show that adding individuals improves their ability to explore the space of solutions in both cases of complexity measures. The swarm-inspired method, combined with a maze environment can provide good ways of dealing with the problem of challenging mazes. Planning to implement a swarm-based approach for supporting the solution of the considered problem we have to ensure the successful integration of the method in maze environments.

As a final remark, it is worth to mention that the relationship between parameter settings and features of difficult mazes is an open question. The more research might prove useful in determining the relationship between parameters and the maze pattern. In the future, a careful analysis will be made of the influence of all major parameters (the visibility parameter, the population size with the number of iterations, a way of choosing exchange points, and so on). It should be mentioned that the tuning and control of algorithm parameters are difficult tasks, and in the case of challenging mazes a careful analysis of the parameter settings would require the use of speed-up techniques or parallel computing. Therefore, one possibility for future research is to use a self-tuning method with a GPU implementation.

Based on our method, it would be interesting to concentrate on various real applications of mazes. Although all experiments were done with the demonstration environment, we believe that this approach may encourage the development of swarm-based approaches to real environments. Therefore, we hope to continue to explore the possibilities of swarm algorithms and studying both the complexity and applications of mazes. Another interesting challenge could be to examine the use of other algorithms, that is, nature-inspired deterministic search metaheuristics, which include e.g., central force optimization [52] or dolphin pod optimization [53].

## Figures and Tables

**Table 1:** Results for five selected independent runs.

**Trial**	**5 Individuals**			
	**Initial**	**Output**	**Number**	**Improve [%]**
1	5202	9295	26	78.68
2	7567	9295	12	22.84
3	3069	9381	19	205.67
4	4407	9065	22	105.7
5	3007	6768	18	125.08
**Trial**	**15 Individuals**			
	**Initial**	**Output**	**Number**	**Improve [%]**
1	4598	13,325	15	189.8
2	4212	14,322	13	240.03
3	5289	13,376	31	152.9
4	7693	14,421	16	87.46
5	8478	16,875	30	99.05
**Trial**	**25 Individuals**			
	**Initial**	**Output**	**Number**	**Improve [%]**
1	8478	19,039	40	124.60
2	4235	17,175	37	305.55
3	11,194	17,459	21	55.97
4	4598	16,946	25	268.55
5	7301	16,614	28	127.56

**Table 2:** Selected statistics after 40 iterations.

Population Size	Complexity				Improve [%]			
	Min	Max	Average	STD	Min	Max	Average	STD
5	6768	9381	7972.11	1239.39	22.84	275.75	137.91	73.68
15	13,325	16,875	14,358.57	1198.08	87.46	240.03	168.55	61.00
25	15,470	19,039	17,207.54	809.63	55.97	329.14	241.09	93.67

**Table 3:** Selected statistics for a comparable number of function evaluations.

Population Size	Complexity				Improve [%]			
	Min	Max	Average	STD	Min	Max	Average	STD
5	6768	13,668	9605.38	1900.71	22.84	441.31	161.09	128.76
15	13,325	16,875	14,634.11	1335.24	87.46	240.03	168.77	63.51
25	15,470	19,039	17,207.54	809.63	55.97	329.14	241.09	93.67

**Table 4:** Selected statistics for a fixed number of iterations.

Population Size	Complexity				Improve [%]			
	Min	Max	Average	STD	Min	Max	Average	STD
5	3.205	3.3836	3.3357	0.069	0.38	17.67	12.01	5.165
15	3.48	3.5862	3.5594	0.039	10.29	15.87	14.06	2.553
25	3.4623	3.6980	3.6053	0.051	12.66	19.48	15.08	2.171

**Table 5:** Selected statistics for a comparable number of function evaluations.

Population Size	Complexity				Improve [%]			
	Min	Max	Average	STD	Min	Max	Average	STD
5	3.205	3.3984	3.3494	0.074	0.38	17.90	12.45	4.988
15	3.48	3.5862	3.5617	0.039	10.34	15.87	14.16	2.432
25	3.4623	3.6980	3.6053	0.051	12.66	19.48	15.08	2.171
